# Treatment effect of nafamostat mesylate in patients with COVID-19 pneumonia: study protocol for a randomized controlled trial

**DOI:** 10.1186/s13063-021-05760-1

**Published:** 2021-11-23

**Authors:** Kyunglan Moon, Kyung-Wook Hong, In-Gyu Bae

**Affiliations:** Department of Internal Medicine, Gyeongsang National University Hospital, Gyeongsang National University College of Medicine, Jinju, Republic of Korea

**Keywords:** COVID-19, Randomized controlled trial, protocol, Nafamostat mesilate, Time to improvement

## Abstract

**Background:**

This study is designed to evaluate the main hypothesis that nafamostat mesilate with standard therapy improves the severity and mortality rate in patients with COVID-19 pneumonia.

**Methods:**

We conduct a randomized, open type, multi-institute/center, 2-group clinical trial with COVID-19 pneumonia patients in Korea. Eighty four patients with COVID-19 pneumonia are randomly assigned to intervention group or control group. Patients in intervention group receive the standard therapy with a dose of 0.1 to 0.2 mg/kg/h (2.4 to 4.8 mg/kg/day) of nafamostat mesilate. Patients in control group receive the standard therapy such as lopinavir/ritonavir, hydroxychloroquine, oxygen therapy, non-invasive and invasive ventilator, antibiotic therapy, renal-replacement therapy, and extracorporeal membrane oxygenation (ECMO). The primary outcome is proportion of patients with clinical improvement as defined by live discharge from hospital or a decline of 2 categories on the seven-category ordinal scale of clinical status, as well as secondary outcome comprised change in National Early Warning Score, duration of hospitalization, incidence of new-non-invasive ventilation or high flow oxygen use or ventilator, mortality at day 28, viral load change, and adverse events.

**Discussion:**

Our study contributes to the establishment of therapeutic strategy in COVID-19 pneumonia by evaluating the therapeutic effect and safety of nafamostat mesilate.

**Trial registration:**

ClinicalTrials.gov NCT04418128. Registered on 5 June 2020.

## Administrative information

**Table:** 

Title {1}	Treatment Effect of Nafamostat Mesylate in Patients With COVID-19 Pneumonia: Open Labelled Randomized Controlled Clinical Trial
Trial registration {2a and 2b}.	ClinicalTrials.gov NCT04418128. Registered on 5th June, 2020.
Protocol version {3}	The current protocol version is 3.1, 11 th June 2020.
Funding {4}	Nafamostat mesylate will be manufactured by SK Chemicals Life Science. National Life Safety Emergency Response Research Program of the National Research Foundation of Korea is funding the trial and 84 patient's recruitment. SK Chemicals Life Science, the manufacturer of nafamostat mesylate, have provided the trial drug used for this trial. The design, management, analysis and reporting of the study are entirely independent of the manufacturers of nafamostat mesylate.
Author details {5a}	Kyunglan Moon, Kyung-Wook Hong, In-Gyu Bae* Authors contributions BIG is the chief investigators; he conceived the study, initiated the study design. HKW contributed to the study design. MK contributed to the study design and development of the proposal. All authors read and approved the final manuscript.
Name and contact information for the trial sponsor {5b}	Trial sponsor: Gyeongsang National University Hospital Address**: 79, Gangnam-ro, Jinju-si, Gyeongsangnam-do, Republic of Korea** Telephone: +82-055-750-8000
Role of sponsor {5c}	Trial sponsors had no role in the study design; collection, management, analysis, and interpretation of the data; writing of the report; and the decision to submit the report for publication.

## Introduction

### Background and rationale {6a}

The COVID-19 epidemic expanded to the whole world since it started from the Wuhan area in China in December 2019. The Republic of Korea experiences a sharp increase in the patient since 24 February 2020. In an analysis of more than 70,000 patients in China, about 15% of them cause severe pneumonia, 5% require treatment in the intensive care unit, and a half of them die of the disease.

There are no proven therapeutics for COVID-19 patients yet. Currently, the treatment with lopinavir/ritonavir, hydroxychloroquine, etc., did not show apparent effect [1], and there are no other drugs that can be applied to patients who get worse even with those drugs or who are severe.

There are research reports that defective innate immunity and accelerated activation of the complement cascade, caused by the SARS-CoV-2, induce rapidly progressing pneumonitis [1, 2].

SARS-CoV-2 is a single-stranded RNA-enveloped virus and contains four structural proteins (S, E, M, and N) [3]. The spike (S) protein of coronaviruses facilitates viral entry into target cells [4]. Entry requires S protein priming by cellular proteases; SARS-S employs the cellular serine protease TMPRSS2 for S protein priming [4]. The previous study reported that, in MERS-CoV, another type of coronavirus, nafamostat blocks TMPRSS2 activity, thereby suppressing membrane fusion between the virus and human cells, consequently inhibiting MERS-CoV infection [5]. Similarly, in SARS-CoV-2, nafamostat blocks viral entry by inhibiting membrane fusion between the outer membrane of the virus and human cells [4, 6, 7]. Nafamostat mesilate has anti-inflammatory effect by inhibition of the complement pathway and inhibition of cytokine production [8].

Our study contributes to the establishment of treatment strategy in patients with COVID-19 pneumonia by evaluating the therapeutic effect of nafamostat mesilate through comparing the improvement in severity and reduction in mortality between the nafamostat mesylate with standard therapy group and standard therapy only group.

## Objectives {7}

### Research hypothesis

The main hypothesis of this study is that it shows a rapid recovery from symptoms and viral suppression in the intervention group.

## Study objective

### Primary objective

The primary outcome of this study will be to evaluate the proportion of patients with clinical improvement as defined by live discharge from hospital or a decline of 2 categories on the seven-category ordinal scale of clinical status.

### Secondary objective

Secondary objective comprised change in National Early Warning Score, duration of hospitalization, incidence of new-non-invasive ventilation or high flow oxygen use or ventilator, mortality at day 28, viral load change, and adverse events.

## Trial design {8}

The trial is designed as a randomized, open labeled, multicenter, two-arm with 1:1 allocation clinical trial in South Korea. This study compares the effect of nafamostat mesylate injection with standard treatment on 42 patients with COVID-19 pneumonia in the intervention group versus 42 patients with COVID-19 pneumonia with standard treatment only in the control group.

## Methods: participants, interventions, and outcomes

### Study setting {9}

This study is a multicenter trial conducted at 10 hospitals in South Korea to generalize the results, and 10 hospitals are located in various cities.

## Eligibility criteria {10}

### Inclusion criteria

1. 18 years old or older
2. Patients who have been confirmed of COVID-19 infection and has evidence for pneumonia
  A. Confirmation of COVID-19 infection by RT-PCR of SARS-CoV-2
  B. Definite diagnosis of new infiltration of the lungs by chest CT scan of chest radiographic inspection

3. Patients who are within 72 h of COVID-19 pneumonia confirmation
4. Patients with 3 (hospitalization, not requiring supplemental oxygen) or higher in seven-category ordinal scale of clinical status
5. Patients who are eligible for diagnosis/evaluation to chest CT scan and related to it
6. Patients should be able to understand the essence of the clinical trial and to submit a written consent document. For the patients who can understand the nature of the research but cannot sign the document, a relative can agree to the study.

### Exclusion criteria

1. Patients who have a record of HIV or AIDS
2. Female patients, either who are pregnant within 6 months before the investigation, who breastfed babies within 3 months before the investigation, or who may get pregnant or breast-feed within 1 month after the investigation is over
3. Patients at high risk of death within 3 days of randomized assignment, by the judge of the investigator
4. Patients with liver cirrhosis with Child-Puch score being B or C
5. Patients who have liver disease abnormalities with ALT or AST > 5 times ULN
6. Patients who can be in danger or who shows clinically important other conditions which may interfere with the evaluation or completion of the test procedure, as the investigator’s opinion
7. Patients who are not appropriate for the test, as the investigator’s opinion
8. Patients who have hypersensitivity to the investigational drug

## Who will take informed consent? {26a}

The principal investigator or assistant researcher with trained research nurse in each hospital explains the purpose, potential benefits, and risks of the study and the right to refuse to participate or withdraw consent at any time and will obtain written consent from patients willing to participate in the trial. Copies of the consent document are provided for all patients involved in the study.

## Additional consent provisions for collection and use of participant data and biological specimens {26b}

Additional biological samples will be obtained to be stored for use in future studies of the pathobiology of COVID-19. A materials consent will be obtained to specifically address the collection of urine, serum, and plasma specimens.

The data collection/request is covered in the original informed consent process for the COVID-19 trial, and a separate consent form for research/donation of human materials administered by the Gyeongsang National University Hospital Institutional Review Board is also received.

Participants will be informed that their withdrawal from the ancillary research is possible.

## Interventions

### Explanation for the choice of comparators {6b}

There is no proven treatment for COVID-19 patients yet, and the standard treatment group is justified as it contains drugs and treatments known to be effective for COVID-19 patients to date. Drugs found to be effective in patients with COVID-19 will be added to standard treatment by protocol modification.

### Intervention description {11a}

Eligible patients will be randomized in equal proportions between standard treatment with nafamostat mesylate and standard treatment only.

Experimental group: nafamostat mesylate plus symptomatic treatment and lopinavir/ritonavir, hydroxychloroquine, antibiotics, non-invasive and invasive ventilation, renal replacement therapy (e.g., CRRT, HD), and ECMO as needed

*Nafamostat injection

- Dosage: the researcher administers a dose of 0.1 to 0.2 mg/kg/h (2.4 to 4.8 mg/kg/day), taking into account the severity and underlying disease of the clinical trial patient.
- Method of administration: nafamostat injection is mixed with 1000 ml of 5% DW infusion, followed by continuous infusion over 24 h.
- Duration of administration: the researcher administers for 10–14 days considering the severity and underlying disease of the clinical trial patient.

Control group: symptomatic treatment and lopinavir/ritonavir, hydroxychloroquine, antibiotics, non-invasive and invasive ventilation, renal replacement therapy (e.g., CRRT, HD), and ECMO as needed

### Criteria for discontinuing or modifying allocated interventions {11b}

Suspension of administration: If the following serious adverse reaction occurs during the administration of the test drug, administration shall be stopped immediately.

1. Symptoms of shock or anaphylaxis
2. Severe hyperkalemia: when there is a change in the electrocardiogram due to hyperkalemia or when the potassium blood concentration is above 7.0 mEq/L
3. Severe hyponatremia: sodium blood concentrations below 120 mEq/L

### Strategies to improve adherence to interventions {11c}

Since the intervention protocol is performed during hospitalization, no adherence reminder is required beyond confirmation of discomfort associated with injectable medications. At the follow-up visit, the participant will be asked questions about any problems encountered after administration of the study medication.

### Relevant concomitant care permitted or prohibited during the trial {11d}

There are no contraindications to use in combination with the permission of the test drug.

### Provisions for post-trial care {30}

The Gyeongsang National University Hospital has insurance to cover for non-negligent harm associated with the protocol. This will include cover for compensation or damages whether awarded voluntarily by the Sponsor or by claims pursued through the courts. Incidences judged to arise from negligence (including those due to major protocol violations) will not be covered by study insurance policies. The liability of the manufacturer of nafamostat mesylate is strictly limited to those claims arising from faulty manufacturing of the commercial product and not to any aspects of the conduct of the study.

When a patient enrolled in the study has discharged, the clinical research coordinator contacts the participants to check for occurring side effects after discharge and provide information on the outpatient follow-up schedule. During outpatient visit including blood tests, adverse events are checked.

## Outcomes {12}

### Primary outcome

The primary outcome is as follows: proportion of patients with clinical improvement as defined by live discharge from hospital or a decline of 2 categories on the seven-category ordinal scale of clinical status [time frame: day 14 and day 28]

*Seven-category ordinal scale of clinical status

1. Not hospitalized with resumption of normal activities;
2. Not hospitalized, but unable to resume normal activities;
3. Hospitalization, not requiring supplemental oxygen;
4. Hospitalization, requiring supplemental oxygen;
5. Hospitalization, requiring nasal high-flow oxygen therapy and/or noninvasive mechanical ventilation;
6. Hospitalization, requiring extracorporeal membrane oxygenation and/or invasive mechanical ventilation;
7. Death.

### Secondary outcome

#### Clinical outcome

1. Time to clinical improvement (TTCI) [time frame: up to 28 days]
  A. Time to clinical improvement (TTCI) was defined as time from randomization to a decline of 2 categories on the seven-category ordinal scale of clinical status or live discharge from the hospital, whichever came first
2. Clinical status assessed by 7-category ordinal scale on days 7, 14, and 28
3. Change in National Early Warning Score (NEWS) from baseline to day 28 [time frame: day 1 through day 28]
*The NEW score has demonstrated an ability to discriminate patients at risk of poor outcomes. This score is based on 7 clinical parameters (respiration rate, oxygen saturation, any supplemental oxygen, temperature, systolic blood pressure, heart rate, level of consciousness). The NEW Score is being used as an efficacy measure
5. Time to National Early Warning Score (NEWS) of ≤ 2 and maintained for 24h 28 [time frame: day 1 through day 28]
6. Duration of hospitalization [time frame: day 1 through day 28] measured in days
7. Duration of new non-invasive ventilation or high flow oxygen use [time frame: day 1 through day 28]
8. Incidence of new non-invasive ventilation or high flow oxygen use [time frame: day 1 through day 28]
9. Duration of new supplement oxygen use [time frame: day 1 through day 28]
10. Incidence of new supplement oxygen use [time frame: day 1 through day 28]
11. Duration of new ventilator or extracorporeal membrane oxygenation (ECMO) use [time frame: day 1 through day 28]
12. Incidence of new ventilator or extracorporeal membrane oxygenation (ECMO) use [time frame: day 1 through day 28]
13. Mortality at day 28 [time frame: day 1 through day 28]. Date and cause of death (if applicable)
14. Time (days) from treatment initiation to death

#### Virological endpoints

1. Proportions of patients with a negative nasopharyngeal swab and sputum sample for SARS-CoV-2 quantitative RT-PCR on days 3, 7, 10, 14, and 21 after starting treatment

2. Viral load change (log10 viral load) of nasopharyngeal swab and sputum sample for SARS-CoV-2 quantitative RT-PCR on days 3, 7, 10, 14, and 21 after starting treatment

#### Safety outcomes

1. Adverse events that occurred during treatment
2. Serious adverse events
3. Premature discontinuation of treatment

## Participant timeline {13}

Nafamostat mesylate protocol schedule of enrolment, interventions, and assessments

**Table:** 

	Screening	Allocation	Post-allocation					Follow-up	
TIMEPOINT**	***-D1***	***D0***	***D1***	***D3***	***D7***	***D10***	***D14***	***D21***	***D28***
**ENROLMENT:**									
**Eligibility screen**	X								
**Informed consent**	X								
**Allocation**		X							
**INTERVENTIONS:**									
*[Nafamostat mesylate]*									
*[Standard therapy]*									
**ASSESSMENTS:**									
*[SARS-CoV RT-PCR]*	X			X	X	X	X		
*[Chest X-ray]*	X			X	X	X	X		
*[Chest CT]*	X						X		
*[Physical examination]*	X								
*[Viral signs]*	X		X	X	X	X	X	X	X
*[Symptoms]*	X		X	X	X	X	X	X	X
*[Seven category ordinary scale]*	X		X	X	X	X	X	X	X
*[NEWS score]*	X				X		X	X	X
*[Blood test]*	X		X	X	X	X	X	X	X
*[Blood collection for research]*			X		X				
*[Adverse reaction]*			X	X	X	X	X	X	X

### Sample size {14}

The sample size was calculated based on the assumption that superiority test (nafamostat treatment is right) of the treatment effect (effect size 10%) between two treatment groups (control group vs. nafamostat-treated group) will be carried out. The superiority margin is set to 0.05 (5%) based on the assumption that the nafamostat-treated group shows higher effect (90.0%) over the control group (80.0%). Nafamostat will be concluded as effective when the nafamostat-treated group shows 5% of higher effect compared to the treatment rate of the control group (80%). A one-sided significance level of 0.05 and power of 80% with equal allocation to two arms would require 42 patients in each arm of the trial.

Basic parameters for the sample size calculation are as follows:

- Significance level, alpha = 0.05 (one-sided test)
- Power = 70%/80%/90%
- The ratio of the control group and the experimental group (nafamostat treatment) patients is 1:1
- Cure rate of the control group: 80%
- The cure rate of the experimental group (nafamostat treatment): 90%
- Superiority margin: 5%
- Expected drop out proportion: 5% (control, experimental each)

### Recruitment {15}

Each center will screen subjects to achieve sample size; screening will continue until the target population is achieved (10 subjects/site). To meet the target sample size, the clinical research associate of each institution identified the difficulty of recruitment and resolved the problem through the entire meeting. In addition, in order to reduce the number of participants allocated to each institution, we contacted other institutions to secure more participating institutions. The enrollment period will extend over 12 months.

## Assignment of interventions: allocation

### Sequence generation {16a}

Nafamostat mesylate with standard therapy group and standard therapy only group were assigned in a 1:1 ratio by block randomization method by institution.

The randomization generation program is as follow: randomization codes are generated using SAS® (SAS Institute, Cary, NC, USA) and assigned to a test group (nafamostat administration + standard treatment) or a control group (standard treatment) according to the generated assignment order.

### Concealment mechanism {16b}

Participants are randomized using an online randomization program, but the allocation concealment is not applicable as this is an open label trial.

### Implementation {16c}

All patients who give consent for participation and who fulfill the inclusion criteria will be randomized. Randomization will be requested by the investigator responsible for recruitment and clinical interviews. The clinical research associate and research nurse access the online program to randomization and then deliver the results to the investigator.

## Assignment of interventions: blinding

### Who will be blinded {17a}

Not applicable as this is an open label trial.

### Procedure for unblinding if needed {17b}

Not applicable as this is an open label trial.

## Data collection and management

### Plans for assessment and collection of outcomes {18a}

Data on demographic information, underlying disease, initial symptoms, diagnosis date, coronavirus Ct value, and laboratory and radiological findings is collected by the clinician. The clinician evaluates the seven-category ordinal scale of clinical status on admission, NEWS score and clinical course complications at hospitalization, treatment progress and clinical results, and side effects by drugs. Medical history is collected within 1 year before screening. In the case of single malignant tumors, medical history within 5 years before screening is collected. Information about underlying disease such as diabetes, hypertension, liver cirrhosis, chronic kidney disease without hemodialysis, end-stage renal disease, chronic heart failure, and lung diseases is collected regardless of period. At each visit, the National Cancer Institute Adverse Events Standard Terminology (NCI CTCAE) version 5.0 should be documented and recorded. The collection period for these reactions is from the start of treatment in the test or control group to day 28.

### Plans to promote participant retention and complete follow-up {18b}

#### Participants retention

Once a participant is enrolled or randomized, the researcher will make every reasonable effort to follow the participants for the entire study period. The research nurse will contact the participants before the scheduled follow-up date to avoid loss of follow-up.

#### Participants withdrawal

The patient has the right to voluntarily discontinue participation in the trial for any reason at any time, and the investigator also has the right to discontinue the patient’s participation in the trial at any time.

Reasons for discontinuing participation in a clinical trial may include, but are not limited to:

- Withdrawal of consent from patients
- Termination of testing or closing of testing institutions
- Non-compliance of patients defined as non-compliance with the clinical trial plan requirements according to the decisions of the tester or client
- Occurrence of serious adverse events

### Data management {19}

#### Data forms and data entry

In this trial, all data will be entered electronically. This may be done at the participating site where the data originated. Original study forms will be entered and kept on file at the participating site. Participant files are to be stored in numerical order and stored in a secure and accessible place and manner. Participant files will be maintained in storage for a period of 3 years after the completion of the study.

#### Data transmission and editing

The data entry screens will resemble the paper forms approved by the committee. Modifications to data written to the database will be documented through either the data change system or an inquiry system. Data entered into the database will be retrievable for viewing through the data entry applications. The type of activity that an individual user may undertake is regulated by the privileges associated with his/her user identification code and password.

#### Data discrepancy inquiries

Additional errors will be detected by programs designed to detect missing data or specific errors in the data. These errors will be summarized along with detailed descriptions for each specific problem in data query reports, which will be sent to the data managers at the C&R research.

## Confidentiality {27}

All study-related information will be stored securely at the study site. All participant information will be stored in locked file cabinets in areas with limited access. All laboratory specimens, reports, data collection, process, and administrative forms will be identified by a coded ID [identification] number only to maintain participant confidentiality. All local databases will be secured with password-protected access systems. Participants’ study information will not be released outside of the study without the written permission of the participant.

## Plans for collection, laboratory evaluation, and storage of biological specimens for genetic or molecular analysis in this trial/future use {33}

To provide a resource for studies of inflammatory markers for COVID-19 pneumonia, white blood cell and plasma will be collected. Because the sample bank is a limited resource, proposals to use it will be evaluated in terms of scientific relevance, significance, and validity as well as the potential impact of the proposed study. The amount and type of material needed will also be considered, and the efficient use of material will be required. Strict confidentiality will be exercised and the information provided to investigators will not contain personal identifiers. Participation in this research is not required for continued participation in the nafamostat-mesylate trial. Although the original model consent form addresses genetic studies, participants will be asked to sign an additional consent form to document their consent to the collection and submission of additional blood samples for storage and future testing. The Global Clinical Central Lab (GCCL) in Yongin City, South Korea, will serve as the processing, aliquotting, and storage facility. Otherwise, the blood will be centrifuged in each institute and stored at sample bank. Plasma will be separated into aliquots and frozen.

## Statistical methods

### Statistical methods for primary and secondary outcomes {20a}

1. Intention-to-treat analysis and per protocol analysis will be conducted respectively, and intention-to-treat analysis will be performed as the main analysis.
2. The categorical variable of the evaluation index will be analyzed using Pearson’s chi-square test, and the continuous variable will be analyzed using ANOVA or Kruskal–Wallis test.
3. The virus removal rate on day 14, one of the secondary outcomes, will be analyzed using Pearson’s chi-square test.
4. Time to clinical improvement will be assessed on all subjects by day 28. If there is no clinical improvement or a case of death by day 28, it will be recorded as day 28, and analyzed by Kaplan–Meier plot and log-rank test.

### Interim analyses {21b}

There is no interim analysis planned yet.

## Methods for additional analyses (e.g., subgroup analyses) {20b}

There is no interim analysis planned yet.

## Methods in analysis to handle protocol non-adherence and any statistical methods to handle missing data {20c}

Outcome data obtained from all randomized participants will be included in the main analyses to prevent attrition bias. To prevent missing data, the researcher, trained research nurse, and research assist will double check the data as well as the recording of reasons for missing data. By replacing missing data with the mean or median value, we plan to analyze with minimal data loss.

## Plans to give access to the full protocol, participant-level data, and statistical code {31c}

No later than 3 years after the collection of the 1-year post-randomization interviews, we will deliver a completely deidentified dataset to trial registries for sharing purposes.

## Oversight and monitoring

### Composition of the coordinating center and trial steering committee {5d}

Not applicable

### Composition of the data monitoring committee, its role and reporting structure {21a}

The Data Monitoring Committee (DMC) has not been established. Although DMC is strongly recommended in other clinical studies with significant safety concerns in which double-blind treatment is assigned, this trial is a phase 2 clinical study with confirmed drug safety and is an open label trial, not a double-blinded study. In the pandemic caused by SARS-COV-2, an effective treatment was not established, and the study was conducted in consideration of the exceptional situation in which a therapeutic agent is urgently needed.

### Adverse event reporting and harms {22}

In our study, an adverse event will be defined as medical occurrence in a subject with regard to the possibility of a causal relationship. Adverse events will be collected after the subject has provided consent and enrolled in the study. If a subject experiences an adverse event after the informed consent document is signed (entry) but the subject has not started to receive study intervention, the event will be reported as not related to study drug. All adverse events occurring after entry into the study and until hospital discharge will be recorded. An adverse event that meets the criteria for a serious adverse event (SAE) between study enrollment and hospital discharge will be reported to the Gyeongsang National University Hospital IRB [Institutional Review Board] as an SAE. A serious adverse event for this study is any untoward medical occurrence that is believed by the investigators to be causally related to study drug and results in any of the following: life-threatening condition (that is, immediate risk of death), severe or permanent disability, prolonged hospitalization, or a significant hazard as determined by the Data Safety Monitoring team. Serious adverse events occurring after a subject is discontinued from the study will not be reported unless the investigators feels that the event may have been caused by the study drug or a protocol procedure. Investigators will determine relatedness of an event to study drug based on a temporal relationship to the study drug, as well as whether the event is unexpected or unexplained given the subject’s clinical course, previous medical conditions, and concomitant medications.

### Frequency and plans for auditing trial conduct {23}

Auditing is considered both overall and for each recruiting center when participants are enrolled. Audits can be done by exploring the trial dataset or performing site visits by C&R research which is a research assist company with independent to investigators. The CRA will verify the events and adverse reactions that occur in all the follow-up visits and the rest of the variables described in the protocol. The monitoring plan is designed to include at least one initiation and three monitoring visits per year.

### Plans for communicating important protocol amendments to relevant parties (e.g., trial participants, ethical committees) {25}

In April 2020, version 2.0, which is initial version of the trial protocol, was submitted to the Ministry of Food and Drug Safety in Korea (MFDS). MFDS approval was granted on April 17 2020. In May 2020, an amendment (version 3.0) was submitted to the MFDS. Protocol changes in this amendment included the following: the contents of patients without standard treatment (e.g., emergency) were revised by referring to the definition of vulnerable subjects in the clinical trial management criteria, addition of serious adverse events (including LFT abnormalities) to the discontinuation criteria, NEWS (National Early Warning Score) added in clinical data collection, revision of the definition of adverse events and actions to be taken in the event of an adverse reaction, and addition of information regarding the storage institution and disposal method of the consent form for human materials. MFDS approved version 3.0 on May 28, 2020. In June 2020, a minor amendment (version 3.1) was submitted to the MFDS. Protocol changes in this amendment included the following: change schedule in the confirmation of virological endpoints through SARS-CoV-2 quantitative RT-PCR. MFDS approved version 3.1 on June 11, 2020. Patient recruitment commenced in April 2020, and the last patient will be recruited to the trial in April 2021. The last visit of the last patient is anticipated to occur in April 2021.

### Dissemination plans {31a}

There are plans for investigators and sponsor to communicate trial results to participants and public via publication of article.

## Discussion

There is lack of proven therapeutics for COVID-19 patients yet. Although the replication pathways of SARS-CoV-2 are still uncertain, replication of SARS coronavirus is mediated by cell surface and endosomal pathways using a protease, the type II transmembrane protease [9, 10]. Nafamostat mesilate shows an anti-viral effect by an inhibition serine protease, which is required for the host membrane fusion of viral envelope protein. In vitro experiments showed that the drug is effective in MERS-CoV, influenza virus, and 2019-nCoV [3–5, 11].

There are studies that have shown that cytokines are elevated in COVID-19 patients [12, 13]. Nafamostat mesilate has anti-inflammatory effect by inhibition of the complement pathway and inhibition of cytokine production [8].

According to our knowledge, this study is the first randomized clinical trial in the world to evaluate the effect of nafamostat mesilate on the COVID-19 pneumonia. Our study contributes to the establishment of therapeutic strategy by evaluating the therapeutic effect and safety of nafamostat mesilate.

## Trial status

The current protocol version is 3.1, 11 June 2020. The study started on 17 April 2020. The first patient enrolled on 7 July 2020. An approximate date on which recruitment will be completed will be 24 April 2021.
